# Two Cases of Primary Hyperparathyroidism During Pregnancy and Post-Partum

**DOI:** 10.1210/jcemcr/luaf177

**Published:** 2025-08-14

**Authors:** F N U Varsha, Michael Grimes, Gayatri Jaiswal, Patricia Bononi

**Affiliations:** Division of Endocrinology, Department of Medicine, Allegheny Health Network, Pittsburgh, PA 15212, USA; Division of Endocrinology, Department of Medicine, Allegheny Health Network, Pittsburgh, PA 15212, USA; Division of Endocrinology, Department of Medicine, Allegheny Health Network, Pittsburgh, PA 15212, USA; Division of Endocrinology, Department of Medicine, Allegheny Health Network, Pittsburgh, PA 15212, USA

**Keywords:** primary hyperparathyroidism, hypercalcemia in pregnancy, parathyroidectomy during pregnancy

## Abstract

Primary hyperparathyroidism (PHPT) is rare in pregnancy and poses diagnostic challenges due to overlapping symptoms. This case series highlights diagnostic and management challenges in pregnant patients. Case 1: A 42-year-old woman at 33 weeks’ gestation exhibited severe nausea and fatigue. Laboratory testing revealed elevated calcium 13.2 mg/dL (3.29 mmol/L) (reference range, 8.4-10.3 mg/dL [2.2-2.6 mmol/L]) and parathyroid hormone (PTH) 215 pg/mL (23.89 nmol/L) (reference range, 11-68 pg/mL [SI: 1.6-7.2 pmol/L]). Neck ultrasound identified bilateral parathyroid adenomas and abdominal ultrasound showed polyhydramnios. Parathyroidectomy resulted in calcium drop to 9.5 mg/dL (2.27 mmol/L) and PTH to 12 pg/mL (1.33 pmol/L). She delivered a healthy infant. Case 2: A 39-year-old woman at 39 weeks’ underwent a cesarean delivery due to transverse fetal lie. She had high prepartum calcium of 14.2 mg/dL (3.55 mmol/L) and PTH 319 pg/mL (33.81 pmol/L). Post pregnancy, bilateral neck exploration and left inferior parathyroid excision decreased calcium to 8.9 mg/dL (2.22 mmol/L) and PTH to 16.5 pg/mL (1.75 pmol/L). These cases highlight that symptom severity—not just calcium level—should guide parathyroidectomy. Third-trimester surgery can be safely performed when symptomatic; asymptomatic patients may be managed expectantly. Early recognition and individualized management optimize maternal and fetal outcomes.

## Introduction

Primary hyperparathyroidism (PHPT) during pregnancy is rare, affecting 1 in 1000 pregnancies [1]. PHPT is commonly caused by solitary parathyroid adenoma (80%-85% of cases), hyperplasia, or in rare cases, carcinoma [2]. Clinical manifestations include nausea, vomiting, fatigue, constipation, and cardiac arrhythmia [3], but 80% of PHPT cases are asymptomatic [2]. Due to enhanced screening and proactive management of osteoporosis, the recorded incidence of PHPT has increased; however, it is rarely diagnosed during pregnancy [1]. Standard imaging modalities, like Technetium (99 mTc) sestamibi, positron emission tomography–computed tomography (PET-CT) are contraindicated in pregnancy because of safety concerns. Symptoms can overlap with normal pregnancy signs, further complicating diagnosis. This case series on PHPT during pregnancy and post partum outlines the need for clear diagnostic guidelines for diagnosis and management in pregnant and lactating patients.

PHPT can often go undiagnosed during pregnancy due to pregnancy symptoms and physiological changes masking PHPT presentation. Pregnancy and lactation induce changes in calcium metabolism to ensure adequate calcium supply for fetal and infant development. The placenta and breasts release parathyroid hormone–related peptide (PTHrP) for calcium transfer to the fetus and breast milk, while 1,25-dihydroxyvitamin D increases intestinal absorption of calcium [4]. These factors elevate renal calcium clearance, leading to hypercalciuria. This, along with lower albumin due to plasma expansion, can mask hypercalcemia and delay diagnosis [4]. However, albumin-corrected and ionized calcium remains stable, and should guide clinical decisions [4]. After delivery, calcitriol levels normalize while PTHrP increases further, and data on PTH are inconsistent [5].

While many mild cases can be managed conservatively, untreated PHPT can lead to severe maternal and fetal complications, including hypercalcemic crises, pancreatitis, nephrolithiasis, hyperemesis gravidarum, and preeclampsia [2]. Hypercalcemia may also cause intrauterine growth restriction, preterm labor, miscarriage, and neonatal issues, including hypocalcemia and seizures, indicating a direct correlation between the degree of hypercalcemia and severity of complications [3]. Polyhydramnios has also been associated with PHPT, likely due to osmotic polyuria in the fetus [6].

Management of PHPT during pregnancy involves both maternal and fetal monitoring. For mild cases, conservative measures, including adequate hydration, are sufficient. Severe cases require surgery, particularly when the patient's calcium levels are greater than 2.85 mmol/L (>11.42 mg/dL) and/or their ionized calcium levels are greater than 1.45 mmol/L (>5.81 mg/dL) [7]. A multidisciplinary approach is crucial for managing symptomatic or severe PHPT during pregnancy to optimize maternal and fetal outcomes and minimize the risk of pregnancy-related and postpartum complications.

Here, we present two cases of PHPT in pregnant patients.

## Case Presentations

### Case 1

A 42-year-old woman G1P0 at 33 weeks' gestation presented with nausea, daily emesis, generalized weakness, increased thirst, and hypercalcemia. Antenatal ultrasound demonstrated mild polyhydramnios. Her family history was negative for hyperparathyroidism, multiple endocrine neoplasia (MEN) syndromes, or other endocrinopathies. She denied taking calcium, vitamin D supplements, or thiazide diuretics. A physical examination revealed a blood pressure of 131/71 mm Hg and a heart rate of 98 beats per minute. She weighed 79.8 kg (176 pounds), with a height of 5 feet 5 inches (∼1.65 m) and a body mass index (BMI) of 33.25. The examination also revealed dry oral mucosa and an enlarged and nodular thyroid gland without cervical adenopathy, but no tremors were observed in the patient's outstretched hands.

### Case 2

A 39-year-old woman at 39 weeks’ of gestation with 2 prior successful pregnancies presented to our institution for cesarean delivery due to fetal transverse lie. She reported an uneventful course throughout the pregnancy. Medications included prenatal vitamins, and she denied additional calcium or vitamin D supplements. She did not have prior hypercalcemia and denied having a family history of hypercalcemia, MEN syndromes, or other endocrinopathies. A physical examination revealed a blood pressure of 109/71 mm Hg and a heart rate of 74 beats per minute. She weighed 86.2 kg (190 pounds) with a height of 5 feet 2 inches (∼1.57 m) and a BMI of 34.76. The examination demonstrated normal mucous membranes, a normal thyroid gland, a regular heart rate, and no tremors in the outstretched hands.

## Diagnostic Assessment

### Case 1

Differential diagnoses included PHPT, familial hypocalciuric hypercalcemia, MEN syndrome, vitamin D intoxication, milk-alkali syndrome, hyperemesis gravidarum, and gestational diabetes mellitus. Laboratory evaluation revealed significant hypercalcemia with normal renal function and 25-hydroxyvitamin D (25[OH]D) levels, elevated PTH levels, and normal serum glucoses and thyroid function (Table 1). The findings confirmed diagnosis of PHPT.

**Table 1.** Blood and urine test results for a pregnant patient in her early 40s who was diagnosed with and surgically treated for primary hyperparathyroidism.

Lab	Upon admission	During hospitalization	At follow up	Reference range
Sodium	139 mEq/L (139 mmol/L)	137 mEq/L (137 mmol/L)	135 mEq/L (135 mmol/L)	136-145 mEq/L (136-145 mmol/L)
Creatinine	0.32 mg/dL (28.33 µmol/L)	0.45 mg/dL (39.78 µmol/L)	0.31 mg/dL (27.44 µmol/L)	0.5-0.9 mg/dL (53-97 µmol/L)
Glucose	205 mg/dL (11.38 mmol/L)	103 mg/dL (5.72 mmol/L)	123 mg/dL (6.83 mmol/L)	70-99 mg/dL (3.88-5.5 mmol/L)
Calcium	13.2 mg/dL (3.29 mmol/L)	12.2 mg/dL (3.05 mmol/L)	9.1 mg/dL (2.27 mmol/L)	8.4-10.3 mg/dL (2.2-2.6 mmol/L)
Ionized calcium	7.08 mg/dL (1.77 mmol/L)	6.68 mg/dL (1.67 mmol/L)	4.88 mg/dL (1.22 mmol/L)	4.5-5.3 mg/dL (1.13-1.32 mmol/L)
Albumin	3.7 g/dL (37 g/L)	3.4 g/dL (34 g/L)	3.5 g/dL (35 g/L)	3.5-5.2 g/dL (35-50 g/L)
Phosphorus	2.1 mg/dL (0.068 mmol/L)	2.6 mg/dL (0.84 µmol/L)	2.7 mg/dL (0.87 µmol/L)	2.5-4.5 mg/dL (0.81-1.45 mmol/L)
Magnesium	1.4 mg/dL (0.0415 mmol/L)	1.6 mg/dL (79.36 µmol/L)	1.4 mg/dL (69.44 µmol/L)	1.6-2.6 mg/dL (0.75-1.05 mmol/L)
Thyroid stimulating hormone	1.54 μIU/mL (1540 mIU/L)	N/A		0.7-1.9 μIU/mL (0.4-4.0 mIU/L)
Parathyroid hormone	215 pg/mL (23.89 nmol/L)	215 pg/mL (23.89 pmol/L)	12.0 pg/mL (1.33 pmol/L)	11-68 pg/mL (1.6-7.2 pmol/L)
25(OH)D	26.8 ng/mL (67 nmol/L)			30-100 ng/mL (70-130 nmol/L)
24 hour urine Calcium and Creatinine	624 mg/24 h (15.56 mmol/d)			100-250 mg/24 h (2.5-7.5 mmol/d)
Lipase	31 U/L			6-75 U/L

Abbreviations: N/A, data not available, 25(OH)D=25-hydroxyvitamin D.

**Figure 1.**
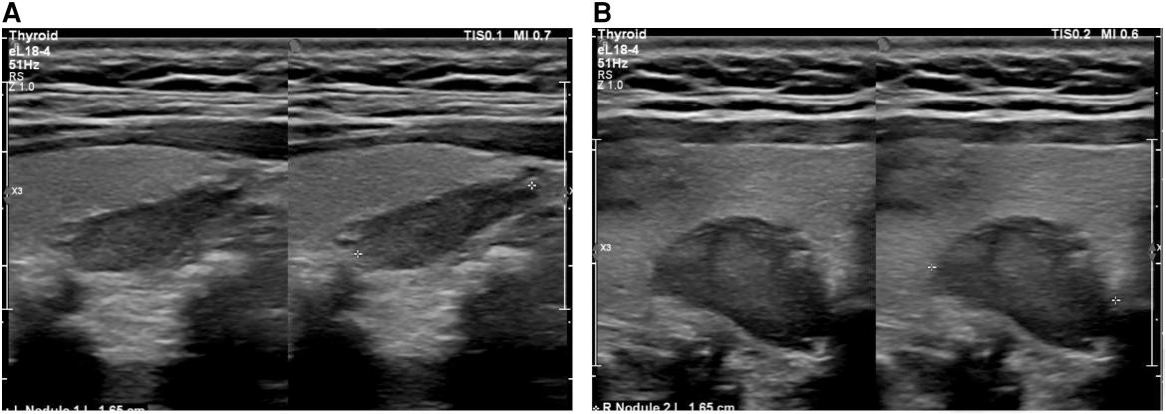
(Case 1): thyroid ultrasound demonstrating bilateral parathyroid adenomas. A, The transverse image of left side of neck showing a well-defined, hypoechoic nodule posterior to the left thyroid lobe, measuring 1.7 cm, suggestive of parathyroid adenoma. B, The transverse image of right side of neck showing a similar hypoechoic nodule posterior to the right thyroid lobe, also measuring approximately 1.7 cm, consistent with parathyroid adenoma.

The patient was treated with intravenous normal saline at 150 mL/hour. A thyroid ultrasound identified bilateral solid hypoechoic nodules posterior and inferior to both thyroid lobes, each measuring 1.7 cm, suggestive of bilateral parathyroid adenomas (Fig. 1). Additionally, a 1.6-cm solid, hypoechoic nodule with regular borders and no calcifications was noted in the right thyroid lobe. Fine-needle aspiration (FNA) of the nodule revealed Bethesda III pathology.

### Case 2

The patient's preoperative laboratory tests indicated hypercalcemia, with a serum calcium level of 14.2 mg/dL (SI: 3.55 mmol/L), along with an elevated PTH levels and low 25(OH)D levels (Table 2), findings confirming PHPT. She delivered a healthy female infant weighing 3660 g (8 pounds, 1.1 ounces). Neonatal calcium was documented as normal at birth, but specific values are unavailable.

**Table 2.** Laboratory Tests

Lab	Values at admission	Values at follow-up	Reference range
Sodium	137 mEq/L (137 mmol/L)	139 mEq/L (139 mmol/L)	136-145 mEq/L (136-145 mmol/L)
Creatinine	0.45 mg/dL (39.78 µmol/L)	0.69 mg/dL (61 µmol/L)	0.5-0.9 mg/dL (53-97 µmol/L)
Calcium	14.2 mg/dL (3.55 mmol/L)	8.9 mg/dL (2.22 mmol/L)	8.4-10.3 mg/dL (2.2-2.6 mmol/L)
Glucose	130 mg/dL (7.2 mmol/L)	84 mg/dL (4.6 mmol/L)	70-99 mg/dL (3.88-5.5 mmol/L)
Ionized calcium	7.66 mg/dL (1.91 mmol/L)	N/A	4.5-5.3 mg/dL (1.13-1.32 mmol/L)
Albumin	3.5 g/dL (35 g/L)	3.9 g/dL (39 g/L)	3.5-5.2 g/dL (35-50 g/L)
Phosphorus	1.9 mg/dL (0.614 mmol/L)	4.6 mg/dL (1.48 mmol/L)	2.5-4.5 mg/dL (0.81-1.45 mmol/L)
Magnesium	1.5 mg/dL (0.617 mmol/L)	1.8 mg/dL (0.76 mmol/L)	1.6-2.6 mg/dL (0.75-1.05 mmol/L)
Thyroid stimulating hormone	0.7 μIU/mL (0.7 mIU/L)		0.7-1.9 μIU/mL (0.4-4.0 mIU/L)
Parathyroid hormone	319 pg/mL (33.81 pmol/L)	16.5 pg/mL (1.75 pmol/L)	11-68 pg/mL (1.6-7.2 pmol/L)
25(OH)D	11 ng/mL (27.46 nmol/L)		30-100 ng/mL (70-130 nmol/L)
24 h urine Calcium and Creatinine	444 mg/24 h (11.1 mmol/d)		100-250 mg/24 h (2.5-7.5 mmol/d)
Lipase	29 U/L		6-75 U/L
PTHrP	< 28 pg/mL (< 2 pmol/L)		< 28 pg/mL (< 2 pmol/L)
Calcitonin		<2 pg/mL (<0.58 pmol/L)	< 5 pg/mL (< 1.46 pmol/L)
Gastrin		24 pg/mL (7.08 pmol/L)	< 100 pg/mL (< 30 pmol/L)
Serum metanephrines		<25 pg/mL (<0.127 pmol/L)	0-88 pg/mL (< 0.5 pmol/L)
Serum Normetanephrines		64.3 pg/mL (0.38 pmol/L)	0-210 pg/mL (< 0.2 pmol/L)

Abbreviations: N/A, data not available, 25(OH)D=25-hydroxyvitamin D, PTHrP=parathyroid hormone–related peptide.

**Figure 2.**
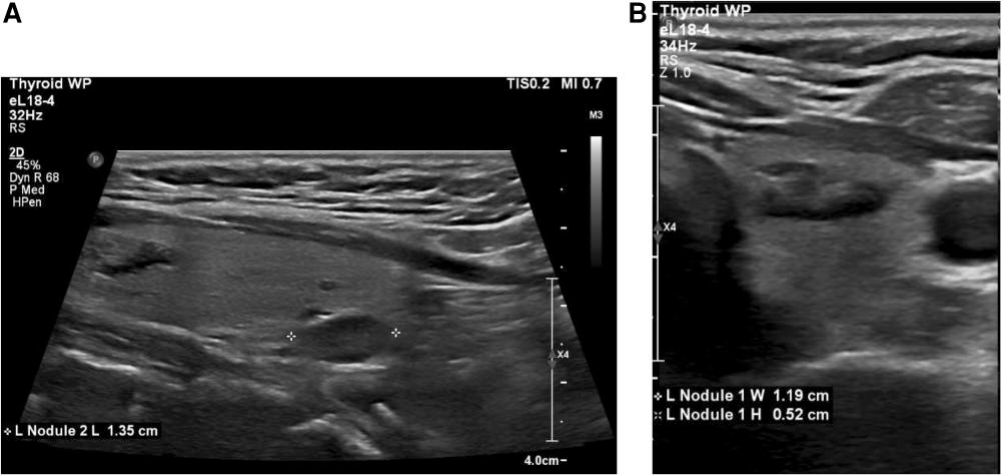
(Case 2): thyroid ultrasound demonstrating bilateral thyroid nodules. A, Transverse image of left thyroid lobe showing a solid, isoechoic nodule in the left superior thyroid pole, measuring 1.2 cm. B, Transverse image of left thyroid lobe showing a solid, hypoechoic nodule in the left inferior thyroid pole, measuring 1.4 cm.

A postdischarge thyroid ultrasound revealed a 1.4-cm solid hypoechoic nodule in the lower pole of the left thyroid lobe with no calcifications and a 1.2-cm solid isoechoic nodule in the upper pole of the left thyroid lobe (Fig. 2). FNA was performed at an outside surgical center at the surgeon's discretion, which is not a recommended approach due to the risk of parathyromatosis [8]. Cytology revealed clusters of benign parathyroid tissue, positive chromogranin staining, and negative staining for synaptophysin, TTF-1, and thyroglobulin. A sestamibi scan failed to localize the parathyroid adenoma. The patient was screened for MEN syndromes; pheochromocytoma was ruled out with a serum metanephrines level of less than 25 pg/mL (SI: <0.127 pmol/L) and serum normetanephrines level of 64.3 pg/mL (SI: 0.38 pmol/L). Calcitonin and gastrin levels were normal (see Table 2).

## Treatment

### Case 1

Following diagnosis, the patient was immediately started on intravenous normal saline at 150 mL/hour. A multidisciplinary team involving endocrinology, gynecology, and ear, nose, and throat surgery recommended semiurgent surgical intervention. Given her age, the patient underwent screening for MEN syndromes including preoperative screening for pheochromocytoma. Fractionated metanephrines were not performed due to time constraints; however, magnetic resonance imaging of the adrenal glands without contrast showed normal adrenal anatomy. The patient underwent parathyroidectomy at 33 weeks of gestation, excising 2 out of 4 parathyroid glands and a concurrent right hemithyroidectomy was performed due to concern for MEN syndrome.

### Case 2

The patient was initiated on intravenous normal saline at 150 mL/hour and ergocalciferol 50 000 units weekly. A few weeks after delivery, the patient underwent bilateral neck exploration and intraoperative identification of radioactive uptake using Technetium-99m and gamma probe localization, leading to the successful resection of the left inferior parathyroid gland.

## Outcome and Follow-up

### Case 1

Ten minutes after excision of the right superior gland, the patient's PTH levels decreased 68% from 274 to 86.5 pg/mL (SI: 9.21 pmol/L). After the left inferior gland excision, her PTH levels further decreased to 18.2 pg/mL (SI: 1.94 pmol/L; 93% reduction overall).

**Figure 3.**
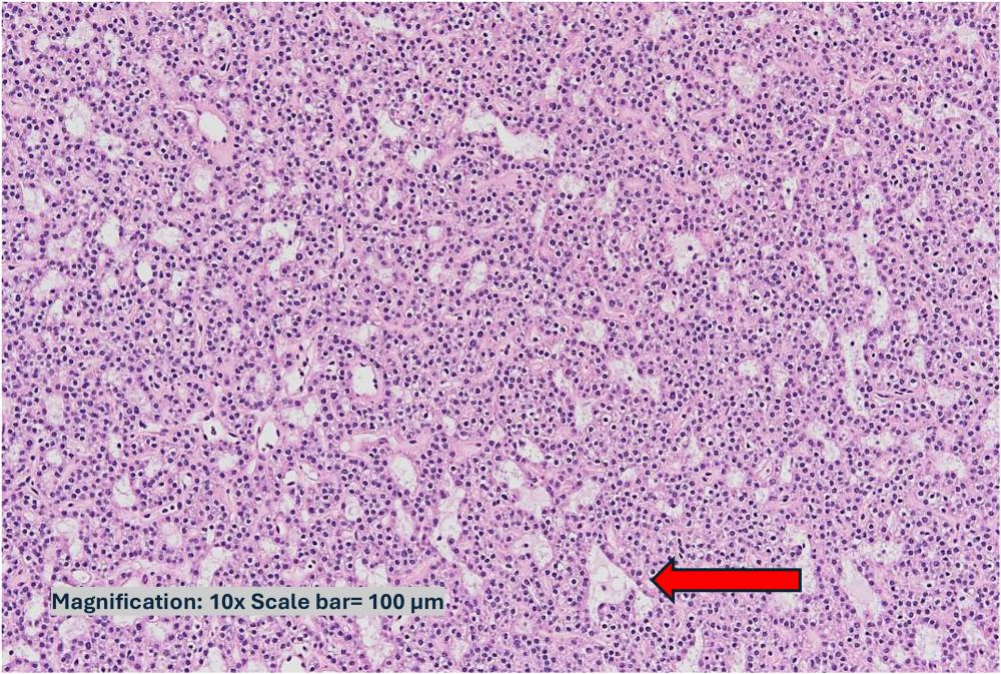
(Case 1): histopathology of parathyroid adenoma. High-power view of a hypercellular parathyroid gland. *The stromal adipocytes (highlighted in arrow) are markedly reduced and, in some foci, completely absent.

Pathology confirmed parathyroid adenomas, with the right gland weighing 1.019 g and the left parathyroid gland weighing 0.2 g (Fig. 3). Hemithyroidectomy revealed a benign pathology.

Postoperatively, the patient's nausea and vomiting resolved, and her calcium and PTH levels remained within the normal range (see Table 1). At 39 weeks, she delivered a healthy infant via cesarean delivery, with a birth weight of 3590 g (7.91 pounds). Neonatal calcium was documented as normal at birth, but specific values are unavailable.

### Case 2

**Figure 4.**
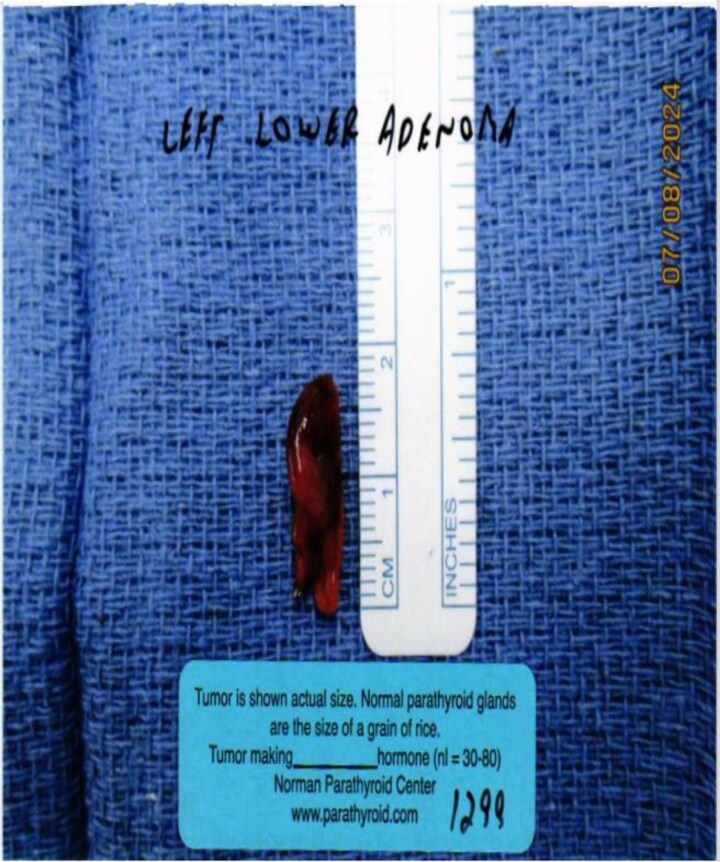
(Case 2): gross specimen of parathyroid adenoma. Gross examination reveals a well circumscribed, reddish-brown soft mass measuring 1.8 cm in length, consistent with a parathyroid adenoma.

Gross examination confirmed parathyroid adenoma (Fig. 4). The other 3 parathyroid glands were biopsied, and histopathologic examination revealed normal parathyroid tissue. The patient's PTH levels dropped to 16.5 pg/mL (SI: 1.75 pmol/L), and she had normalized calcium levels (see Table 2).

## Discussion

Early detection of PHPT helps minimize risks both to the patient and the fetus and supports treatment decisions. Routine calcium screening at least once during pregnancy is recommended to facilitate early detection of PHPT [7]. Persistent hypercalcemia with elevated or inappropriate PTH is a hallmark of PHPT. Differentiating PHPT from familial hypocalciuric hypercalcemia is essential, though it can be challenging due to pregnancy-induced changes in calcium excretion (absorptive hypercalciuria). A 24-hour urinary calcium excretion, along with prepregnancy data and family history, aids in this distinction. In cases where surgery is required, the preoperative evaluations should include screening for pheochromocytoma and MEN syndromes to avoid perioperative complications.

When calcium screenings indicate a potential for PHPT, the next step is imaging. Standard localization imaging modalities, like Technetium (99 mTc) sestamibi, PET-CT are contraindicated due to placental transfer, and alternative approaches such as ultrasound are preferable, followed by bilateral neck exploration and intraoperative PTH monitoring if ultrasound findings are inconclusive [4, 9].

Once diagnosed, treatment may include medications or surgery. Calcitonin may be used in acute hypercalcemia, though its safety remains uncertain during pregnancy [5]. Likewise, bisphosphonates and denosumab are avoided in pregnant and lactating patients due to teratogenic risks. Both cinacalcet and bisphosphonates affect bone and mineral metabolism in infants and therefore are not recommended during lactation. Surgery is typically performed during the second trimester to minimize risks. Surgical approaches use a minimally invasive parathyroidectomy for an adenoma, and bilateral exploration is performed in hereditary forms [7]. Postoperatively, monitoring for hypocalcemia in the mother and potential neonatal hypocalcemia is essential [5, 7].

Our patients were both successfully treated with surgery and delivered healthy infants. In case 1, the patient presented with severe hypercalcemia and was treated with surgery at 33 weeks' gestation to prevent complications. In case 2, the patient was asymptomatic despite calcium levels of 14.2 mg/dL (3.55 mmol/L). Her diagnosis was made at the time of cesarean delivery, and she underwent parathyroid surgery about 2 months post partum. Compared to published case series of PHPT in pregnancy, our two cases underscore the importance of a systematic, protocol-based approach. While many reported cases defer imaging or rely on delayed sestamibi scans post partum, we used ultrasound as a first-line diagnostic tool during pregnancy, which localized parathyroid adenomas without radiation exposure. This facilitated timely surgical planning—one in the third trimester and one soon after delivery—preventing maternal and neonatal complications often described in delayed or undiagnosed cases. Furthermore, our deliberate exclusion of confounding etiologies in these young patients (such as familial hypocalciuric hypercalcemia and MEN syndrome workup) distinguishes our approach from others in the literature, where such evaluations are inconsistently reported. One of our cases was asymptomatic, identified through biochemical screening, illustrating the value of heightened clinical suspicion even in the absence of overt symptoms. These factors set our cases apart and demonstrate how ultrasound can be both effective and safe in guiding early diagnosis and management of PHPT during pregnancy. Our outcomes reinforce the need for standardized diagnostic pathways to reduce delays and complications in this vulnerable population.

## Learning Points

- These cases highlight that symptoms severity—not just calcium level—should guide timing of parathyroidectomy in pregnancy.
- Third-trimester surgery can be safely performed when symptoms are significant.
- While asymptomatic mild hypercalcemia can be managed expectantly until post partum, such patients as seen in case 2 should be carefully monitored at the time of delivery and during the postpartum period to mitigate complications.
- Early recognition and individualized management are key to optimizing maternal and fetal outcomes.
- This case series highlights the need for timely biochemical testing, pregnancy-safe imaging, and multidisciplinary collaborations to guide treatment decisions.

## Data Availability

Original data generated and analyzed during this study are included in this published article.

## Abbreviations

BMI: body mass index
FNA: fine-needle aspiration
MEN: multiple endocrine neoplasia
PET-CT: positron emission tomography–computed tomography
PHPT: primary hyperparathyroidism
PTH: parathyroid hormone
PTHrP: parathyroid hormone–related peptide
